# Mechanical Recycling of a Short Carbon Fiber Reinforced Polyamide 6 in 3D Printing: Effects on Mechanical Properties

**DOI:** 10.3390/polym18010027

**Published:** 2025-12-22

**Authors:** Marco Zanelli, Giulia Ronconi, Nicola Pritoni, Andrea D’Iorio, Monica Bertoldo, Francesco Mollica, Valentina Mazzanti

**Affiliations:** 1 Department of Engineering, University of Ferrara, Via Saragat 1, 44122 Ferrara, Italy; marco.zanelli@unife.it (M.Z.);; 2 Department of Chemical, Pharmaceutical and Agricultural Sciences, University of Ferrara, Via L. Borsari 46, 44121 Ferrara, Italy

**Keywords:** additive manufacturing, Onyx, sustainability, interfacial adhesion, mechanical properties

## Abstract

Mechanical recycling of Fused Deposition Modeling 3D printing materials is very attractive for the circular economy. In this paper, the tensile properties of a virgin and a one-time-recycled short carbon fiber reinforced polyamide, coming from 3D printing scrap and failed parts, were evaluated. Anisotropy was taken into account properly by using characterization methods that are typical of composites. Rheological properties were obtained with a parallel plate rheometer in oscillatory mode, and thermal properties were investigated based on thermogravimetric analysis and differential scanning calorimetry. A decrease in the average molecular weight of the recycled material, indicated by the rheological measurements, induced brittleness. Nevertheless, the stiffness and yield strength of the 3D printed parts made with the recycled material were higher than those made with the virgin one. Since this behavior could not be explained based on an increase in crystallinity or a relevant decrease in the void content, a feasible explanation is proposed with an increase of the interlayer and intralayer adhesion quality. In any case, the recycled polyamide filament can be successfully reused in Fused Deposition Modeling 3D printing, even when significant mechanical properties are required, but attention must be paid to a certain decrease in ductility.

## 1. Introduction

Fused Deposition Modeling (FDM) is arguably the most widely used 3D printing technology, especially concerning thermoplastics. The polymeric materials that are more commonly used as filaments are polylactic acid (PLA) [1], acrylonitrile butadiene styrene (ABS) [2,3], and the polyamides (PA) [4]. This latter family is particularly interesting because it comprises polymers with relatively high mechanical and thermal properties that thus can be used for mildly structural applications. Moreover, these thermoplastics may have a good printability and can be easily compounded with reinforcing fibers, such as glass, basalt or carbon, to enhance mechanical performance even more [5,6,7,8].

Thanks to these advantages, 3D-printed reinforced PAs are widely used in various industrial applications. Given the growing attention towards eco-sustainability, a crucial topic that must be investigated is mechanical recycling. Indeed, scrap production is very common in FDM, as it may come from support structures, purge or failed prints, and also end-of-life products may yield feedstock for recycling. One interesting possibility would be to reuse these materials again for 3D printing, since the starting material had been optimized for this manufacturing technology. The recycling procedure involves a preliminary step of milling, followed by a first heating phase to manufacture the filament and an additional heating phase to print the final part.

Only a few literature studies are devoted to mechanical recycling of reinforced PAs for FDM. Partly, this could be due to some intrinsic difficulties, as pointed out by Mergulhao et al. [9]. These researchers recycled a commercial carbon fiber reinforced PA 6 named “Onyx”, that has been developed specifically for 3D printing by Markforged (Waltham, MA, USA). This material was successfully milled and extruded as a filament, but the printing quality was too low; therefore, the main result that was obtained is that the recycled filament loses stiffness and strength in comparison with the virgin one.

The same material was successfully recycled and printed by Ding et al. [10] and Lohr et al. [11]. In particular, Ding et al. [10] obtained a recycled filament by thermally straightening a knitted 3D printed structure. This had the clear advantage of avoiding the milling phase and led to an improvement in the printability of the material. Interestingly, the tensile properties of the printed recycled material were better, both in terms of stiffness and strength, if compared to printed virgin Onyx. An analogous result concerning bending properties was obtained by Lohr et al. [11], who were able to recycle Onyx twice. Notice, though, that the improvement was limited to the first recycling step, while properties decreased in the second recycling. On the other hand, in agreement with the results of Mergulhao et al. [9], filament mechanical properties decreased after the first recycling. A higher number of recycling steps was obtained only in the case of unreinforced PA 12 by Vidakis et al. [12], who were able to reach six successive cycles. This is not surprising, as PA 12 is more stable against degradation than PA 6, due to the smaller frequency of amide groups in the main chain [13]. Again, 3D printed specimen mechanical properties increased with the number of recycling steps, reaching their maximum after four cycles, before decreasing abruptly.

The case of reinforced PA 12 was also tackled by Bandinelli et al. [14], who obtained similar results with a slightly different additive manufacturing technology, Fused Granular Fabrication, that is anyway based on material extrusion like FDM, but starting with pellets rather than filament. The most interesting feature of this paper is the investigation of anisotropy, which is justified by the presence of short carbon fibers [15]. Indeed, in all the other papers discussed previously [10,11,12], only ±45° raster angle specimens were manufactured. In the case of Bandinelli et al. [14], tensile dogbone specimens at 0° raster angle were fabricated to measure longitudinal properties, and transverse properties were studied by loading helically 3D printed tubular specimens in axial tension. In agreement with the other papers [10,11,12], recycled printed material had stiffness and strength that were higher than the virgin one, but interestingly, such a difference was definitely more significant in the transverse direction [14].

The aim of this work is to recycle a short carbon fiber reinforced PA 6 scrap, coming from industrial 3D printers, and to compare it with the virgin material. Onyx was selected for convenience as reference material, because it was specifically developed for industrial 3D printers and is common for ready-to-use parts. Tensile tests at 0°, 90°, and ±45° are employed to characterize the anisotropic in-plane properties of the virgin and recycled materials. These results are supported by rheological and thermal characterization to achieve a better understanding of the effect of recycling on mechanical performance and printability. The main points of novelty of the present paper, in contrast with previous studies, are the complete characterization of anisotropy in the mechanical properties for both virgin and recycled materials, and the proposed explanation for the unexpected differences in such properties.

## 2. Materials and Methods

### 2.1. Materials

The thermoplastic material investigated in this paper is a commercial PA 6 reinforced with 10 wt.% short carbon fibers, whose commercial name is Onyx, and is distributed in the form of 1.75 mm diameter filament directly by Markforged (Waltham, MA, USA). Markforged is primarily a manufacturer of FDM printers, and its devices are restricted to working only with proprietary materials with an unmodifiable set of printing parameters. The main properties of the material used in this study are detailed in the technical datasheet [16] and outlined in Table 1. Throughout this paper, the commercially purchased virgin Onyx will be referred to with “V”, while the recycled one will be indicated with “R”.

### 2.2. Recycling Process

Recycling starts from scraps, which were supplied by Katakem (Catanzaro, Italy) and consisted of very heterogeneous parts, such as supports, purge lines generated at the beginning of each print, and parts that were either not fit for purpose or discarded because of failed printing. As explained earlier, since processing parameters of Markforged printers cannot be modified by the user, the material always undergoes the same thermal history, independent of the particular source. More in detail, the material is always printed with a 0.4 mm nozzle at a temperature of 275 °C onto an unheated garolite surface, covered with a suitable adhesive layer.

The larger scraps were first cut with a circular saw to obtain grindable parts of approximately 100 mm in size. The material was then ground using a single-shaft shredder with three equally spaced blades. The heterogeneous flakes that were obtained were then sieved through a 4 mm mesh, to homogenize the ground material as much as possible. Before being re-processed, the sieved flakes were dried overnight at 85 °C.

**Figure 1:**
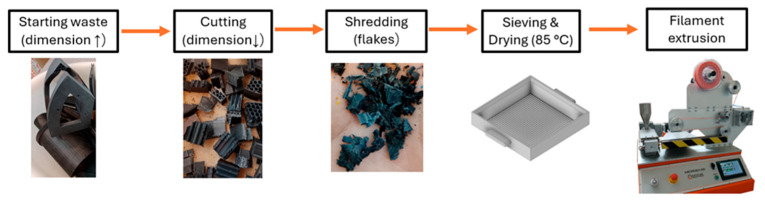
Recycling workflow.

MicroEx 3D single screw extruder, purchased from Eur.Ex.Ma. (Varese, Italy), was used to obtain the filament from the milled scrap. The screw had a diameter of 17.5 mm, an L/D ratio of 22.5 and was operated at 40 RPM. The extruder had three heating zones, two on the barrel and one on the die and were set at 215 °C, 240 °C and 220 °C, respectively. The cooling system utilized a forced-air configuration and comprised three fans. Before pulling and winding, an integrated in-line measuring system provided the filament diameter, which in this case was set at 1.75 mm with a dimensional tolerance of ±0.1 mm. Figure 1 shows the sequence of recycling operations to obtain the R filament, starting from the scrap. Only one recycling run was studied in this paper, as in the second one the material viscosity appeared to be too low to allow extrusion within the required tolerance.

### 2.3. Recycled Material 3D Printing

In order to guarantee good quality and printing reliability, Markforged printers do not allow the use of non-proprietary material and limit the modification of the printing parameters. To this aim, Markforged printers perform a material control routine at the beginning of each print job. As the R material has undergone milling and two heating cycles, it may be sufficiently modified with respect to the V filament, that either the printing quality is not ensured, or the starting control routine does not yield permission to start printing. Moreover, Markforged printers impose an unmodifiable ±45° raster angle on 100% infill printed parts; therefore, it would be impossible to characterize the material in the longitudinal and transverse direction. Consequently, a different printer had to be used for both V and R materials, with the same printing parameters, so that a complete characterization could be performed without processing related differences.

The printer used in this work was an Anet A8 Plus, manufactured by Anet Technology Co. (Shenzen, Guangdong, China), with a 0.6 mm diameter nozzle. To ensure successful printing of the specimens and to have a working environment as controlled and stable as possible, the printer was placed inside a custom-built enclosure. Ultimaker Cura version 5.6.0 was the slicer that was used and the main printing parameters for manufacturing the rheological and mechanical specimens are reported in Table 2. To improve adhesion to the printer’s glass plate, an adhesive from Thought 3D (Qormi, Malta) was used, and an 8-line brim was set from the slicer. Both V and R filaments were dried at 85 °C for 24 h before printing, and the spools were stored in a passive dry box (Markforged, Waltham, MA, USA) containing silica salts during printing.

### 2.4. Voids Measurements

The density $\rho_{samp}$ of rheological and tensile specimens was evaluated before testing. Mass was measured with a precision scale (AdventurerPro AV4102C, Pine Brook, NJ, USA, with ±0.01 g resolution), while a caliper (±0.01 mm resolution) was used to obtain length, while width and thickness were assessed with a micrometer possessing a 0.001 mm resolution (Mitutoyo, Digimatic micrometer, Kawasaki, Japan). At least three measurements were acquired for each dimension. For all printed samples, the percentage of voids v was evaluated as follows [8,17]:

(1) $$v=\left(1-\frac{\rho_{samp}}{\rho }\right)\cdot 100,$$

where the material density ρ is assumed to be 1.2 g/cm^3^, in agreement with the datasheet (Table 1).

### 2.5. Tensile Tests

Short fiber reinforced 3D printed polymeric materials can be regarded and modeled as anisotropic materials like composites [8,15,18,19]. Tensile tests were performed following ASTM D3039 and ASTM D3518 standards [20,21]. The first one is used to measure longitudinal and transverse properties, by printing specimens at 0° and 90° raster angle. In particular, Young’s modulus $E_{1}$, Poisson’s ratio $\nu_{12}$ and longitudinal yield strength $\sigma_{Y}^{0{}^{\circ}}$ were obtained from 0° raster angle, while Young’s modulus $E_{2}$ and transverse yield strength $\sigma_{Y}^{90{}^{\circ}}$ were evaluated from 90° specimens. The second standard is used for measuring the shear properties, i.e., the shear modulus $G_{12}$ and the shear yield strength $\tau_{Y}$, and specimens were printed at ±45° raster angles. For both cases, the samples are rectangular in shape, 110 mm long, 15 mm wide, and 2.3 mm thick.

Tensile tests were carried out at room temperature at a crosshead speed of 5 mm/min using a universal testing machine (INSTRON 34TM-30, INSTRON, Norwood, MA, USA) equipped with a 30 kN load cell. The tests were performed on three samples per raster angle (0°, 90°, ±45°), for a total of nine samples per material. All samples were conditioned at 50% relative humidity and room temperature for at least 8 h prior to testing. For all specimens, Digital Imaging Correlation, or DIC (DANTEC DYNAMICS, Skovlunde, Denmark), was used to measure both longitudinal and transverse strain, as an average value over the analyzed area. To ensure readability by the DIC cameras, the surface of the samples attached to the hot plate was painted with water-based ink.

Following ASTM D3039, $E_{1}$, $E_{2}$, and $v_{12}$ were estimated between 1000 με and 3000 με, while the $G_{12}$ was calculated between 1500 με and 4000 με, as described in ASTM D3518. Yield strength values $\sigma_{Y}^{0{}^{\circ}}$, $\sigma_{Y}^{90{}^{\circ}}$, $\tau_{Y}$ were evaluated as offset strengths, with the value of the offset strain being 10,000 με. Ultimate tensile strain in the longitudinal $\varepsilon_{ul}^{0{}^{\circ}}$ and transverse $\varepsilon_{ul}^{90{}^{\circ}}$ directions were evaluated at complete failure of the specimens. Similarly, the ultimate shear strain $\gamma_{ul}$ was measured from the shear characterization.

### 2.6. Microscopy Analysis

Fracture surfaces and morphological observations of the longitudinal and transverse cross sections of untested tensile specimens were analyzed using a stereomicroscope (Stereo WILD–DFC 420, Leica, Heerbrugg, Switzerland), equipped with LAS software (Version 4.12, Leica, Switzerland) for data and image acquisition. The transversal polished cross section is obtained from the 0° sample, while the longitudinal cross section is taken from the 90° sample. From the cross-sectional view of polished untested samples, the printing quality and the presence and spatial distribution of the voids can be assessed. The procedure was as follows: untested specimens were first cut manually, then cold-embedded in a two-component epoxy resin (Struers S.A.S., Champigny sur Marne Cedex, France), before being lapped using a DAP-V polishing machine (Struers S.A.S., Champigny sur Marne Cedex, France). Specimens were then prepared with wet sandpaper (240, 400, 800, 2400 and 4000 grit) and lapped until a very smooth surface was obtained. The surface was then dried in ambient air and finally observed at the stereomicroscope.

### 2.7. Rheological Analysis

Rheological characterization was performed for both V and R materials with a smooth surface parallel plate rheometer (ARES Rheometric Scientific, New Castle, DE, USA) in dynamic oscillation and strain-controlled mode. The specimens are in the shape of a disc, 25 mm in diameter and 2 mm thick. Prior to testing, a porosity content of less than 10% was verified so that the accuracy of the measurements was ensured.

Three types of rheological experiments were performed: strain, frequency, and time sweeps, all performed at two different temperatures, i.e., 200 and 215 °C. In the strain sweep test, the response of the material to increasing strain amplitudes was measured at constant frequency and temperature for determining the linear viscoelasticity region (LVR), which is important for performing the frequency sweep test correctly. The frequency was set at 1 rad/s, and the strain was evaluated between 1% and 10% at both temperatures. In the frequency sweep test, the response of the material was investigated as a function of frequency at constant strain and temperature. Frequency response was investigated between 0.1 and 100 rad/s. The storage modulus G′ and the loss modulus G″ could be measured as a function of frequency. These values are used to calculate the complex modulus:

(2) $$G^{*}=\sqrt{{\left(G\prime \right)}^{2}+{\left(G\prime \prime \right)}^{2}},$$

from which the complex viscosity $\eta^{*}$ can be obtained, as follows:

(3) $$\eta^{*}=\frac{G^{*}}{\omega },$$

where ω is frequency. The time sweep test was performed at 1 rad/s and 1% strain at both temperatures. This test is useful to evaluate the possibility of degradation by checking the trend of $G^{*}$, G′, and G″ over time at a specific temperature.

### 2.8. Thermal Characterization

Differential scanning calorimetric (DSC) analyses were performed on a DSC 8000, Perkin Elmer Inc. (Waltham, MA, USA) equipped with IntraCooler II cooling device and Pyris software (Version 13.3, PerkinElmer Inc., Waltham, MA, USA) for instrument control, data acquisition, and analysis. The instrument was calibrated for temperature and energy with high-purity indium and lead as standards. All tests were conducted in a nitrogen atmosphere with two thermal cycles at a heating/cooling rate of 10 °C/min between 30 and 250 °C. DSC was used to determine the crystalline fraction and the characteristic temperatures, such as the glass transition temperature $T_{g}$ and the melting temperature $T_{m}$. The crystalline fraction $\chi_{c}$, evaluated as a percentage, was obtained as follows:

(4) $$\chi_{c}=\frac{\Delta H_{m}}{\Delta H_{m0}(1-w_{t})}*100,$$

where $w_{t}$ is the fiber mass fraction, $\Delta H_{m}$ is the experimentally measured melting enthalpy, and $\Delta H_{m0}$ is the melting enthalpy of the fully crystalline polymeric matrix, which for PA 6 was assumed to be equal to 230 J/g [22]. Measurements were carried out on 10–15 mg of material derived from 3D printed samples, and three analyses were performed for both V and R materials. By doing so, it is possible to examine the material in its post-printing state; that is, in the same conditions as those of mechanical tests. An isothermal DSC analysis was also performed on V and R materials using the same instrument. Both tests were carried out on 10 mg of 3D printed samples in nitrogen atmosphere. The samples were heated up quickly (about 1 min) to 200 °C, and the temperature was subsequently maintained for 30 min.

The fiber mass fraction $w_{t}$ was measured through thermogravimetric analysis, or TGA, (PerkinElmer TGA 4000 thermobalance. Waltham, MA, USA), which is useful also to evaluate the thermal stability of the filaments. Three measurements were conducted on 5–10 mg of V material under nitrogen atmosphere (30 mL/min), from 30 to 900 °C at a heating rate of 10 °C/min.

## 3. Results and Discussion

### 3.1. Thermal Characterization Results

**Figure 2:**
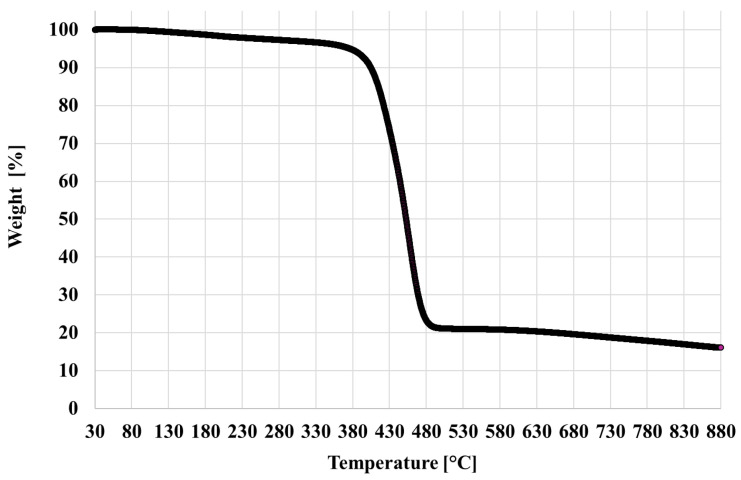
The TGA thermogram for V material.

Concerning TGA results, a minor weight loss (<5%) is visible in the 80–150 °C range and is due to evaporation of the moisture content. Most of the weight drop occurs in the 380–450 °C temperature range leaving an inorganic residual at 900 °C of 14.3 ± 2.1% (Figure 2). Taking into account the initial moisture content, the final mass residue is in agreement with the carbon fiber content declared by the manufacturer and is also in substantial agreement with analogous findings in the literature [23].

**Figure 3:**
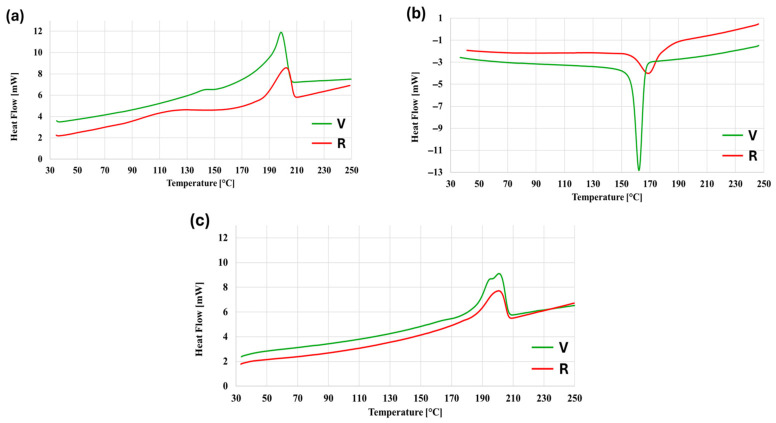
DSC thermograms of V and R, for (**a**) first heating, (**b**) cooling, and (**c**) second heating.

DSC representative curves are reported in Figure 3, for both V and R. The areas of the cooling and melting peaks are always greater for V than those of R, indicating a higher crystalline fraction. From the first heating curves (Figure 3a), two peaks can be observed for both materials: the first one is located at about 120 °C for R and at 140 °C for V. This may be due to several factors, such as the presence of low-order crystalline fractions, which are the result of rapid cooling, but also moisture evaporation, taking the hygroscopicity of the polyamide into account. The main melting peak is located between 190 °C and 210 °C for both materials, but the R material peak appears to shift slightly to higher temperatures. This trend is confirmed also in the cooling curves (Figure 3b), where both materials display a crystallization peak, but that of the R material is markedly shifted to higher temperatures, compared to the one of V. Also, the shape of the peaks is different: the R material has a wider and shorter peak, while that of the V material is narrow and long. This behavior is most probably due to chain scission degradation that occurred during the recycling process, resulting in a wider distribution of molecular weights, with shorter chains providing greater molecular mobility [24,25]. This promotes the kinetics of crystallization of the R material so that the R polymer chains start to crystallize earlier and crystallization proceeds for a wider temperature range. Recycling induced degradation was also found in [26] for the same material. In the second heating (Figure 3c), the secondary peaks at lower temperatures disappear; thus, the principal difference is the shape of the main melting peaks. The one of the V material displays two cusps, which are characteristic of the two crystalline phases of polyamides, α-phase and γ-phase [26,27,28]. The R material has only a single cusp and is located near the second cusp of the V material. Besides the disappearance of the low temperature secondary peaks, the comparison between first and second heating reveals a certain decrease in the main melting peak, especially regarding the V material, while the shape of the peaks of the R material is unchanged between the first and second heating.

The characteristic temperatures and crystalline fractions, as measured from DSC, are listed in Table 3 for both materials and reflect quantitatively what was observed qualitatively in Figure 3. The melting temperatures for the two heating phases are the same, while there is a difference of about 7 °C in the crystallization peaks. The V material has a slightly higher overall crystalline fraction than R. Comparing the first and second heating of both materials, R material crystallinity is the same (around 16%) while for V the very small difference (22% to 19%) may be ascribed to the filament making process, which combines the effects of cooling and stretching immediately after extrusion.

Nevertheless, notice that all crystallinity values are very low; therefore, both materials are essentially amorphous. Usage of amorphous materials is quite common in 3D printing because a relatively high crystallinity usually determines higher volumetric shrinkage, leading to warpage and distortion of the final printed part [29].

### 3.2. Rheological Characterization

**Figure 4:**
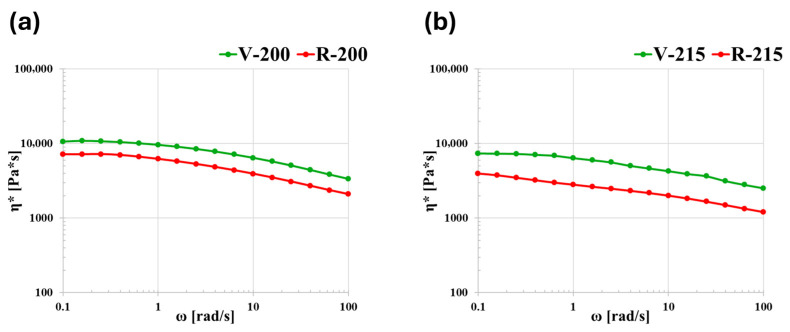
Complex viscosity as a function of frequency for V and R at (**a**) 200 °C and (**b**) 215 °C.

The strain sweep tests ensured that a strain of 1% was sufficient for remaining within the LVR and this value was used for performing all the other rheological measurements. Figure 4 shows the curves obtained from the frequency sweep tests, at 200 °C and 215 °C. For both materials, the trend of complex viscosity $\eta^{*}$ is given as a function of frequency ω. At low frequencies, it is possible to appreciate part of the Newtonian plateau, except for R at 215 °C. The $\eta^{*}$ curve for R is below that of V at both temperatures. This drop in viscosity is related to the decrease in molecular weight due to chain scission material degradation [27,30,31], which confirms the DSC observations.

**Figure 5:**
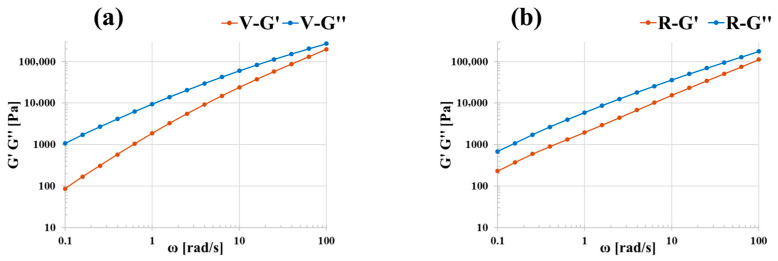
Storage and loss moduli as a function of frequency at 200 °C for (**a**) V and (**b**) R.

The degradation of R can also be observed by plotting G′ and G″ as a function of ω and comparing them with those of V (Figure 5). Since the trend is the same at both temperatures, only the curves at 200 °C are reported. For the R material, the slopes of the curves indicate that the crossover between G′ and G″ shifts at higher frequencies. This is due to a decrease in the relaxation time of the polymer chains (chains relax faster), and this further confirms the degradation of the R material. For the whole range of analyzed frequencies, both materials show a fluid-like behavior (G″ > G′) and both moduli increase with frequency.

**Figure 6:**
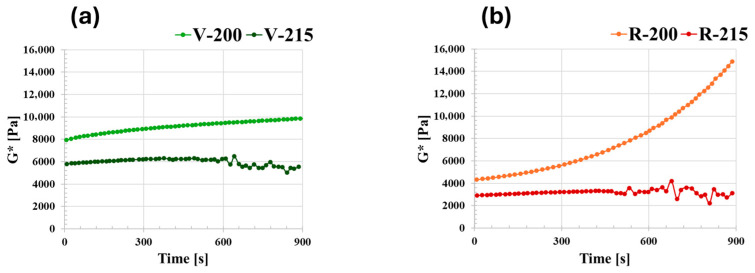
Complex modulus as a function of time at 200 °C and 215 °C for (**a**) V and (**b**) R.

The thermal stability of the materials was evaluated through the time sweep test, at a frequency of 1 rad/s for a total test time of 900 s. Figure 6 shows the trend of $G^{*}$ during the test period. For both materials, the curve at 215 °C is lower than that at 200 °C. The $G^{*}$ curve for V at 200 °C is very slightly increasing, while at 215 °C the material is no longer stable after 600 s (Figure 6a). The R material at 215 °C is stable for about 400 s (Figure 6b), while the trend of $G^{*}$ at 200 °C rises sharply.

**Figure 7:**
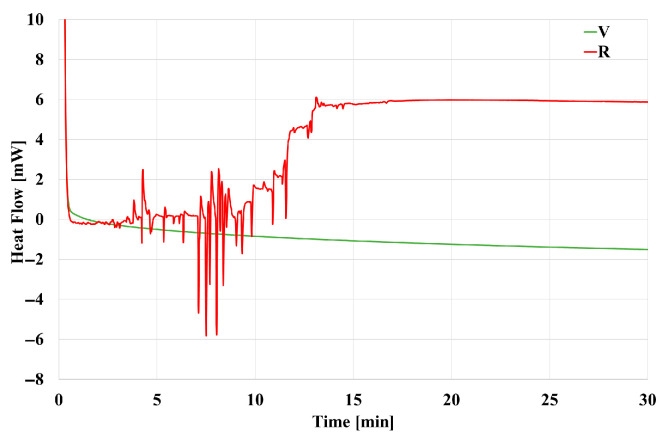
Isothermal DSC thermograms of V and R materials.

In order to explain this behavior, an isothermal DSC scan at 200 °C was performed on both materials (Figure 7). It can be seen that in the same time frame as observed in Figure 6 V remains stable, while the R material undergoes an endothermic transformation, which is not compatible with crystallization. As a consequence, the sharp increase in the $G^{*}$ curve at 200 °C is most probably due to a degradation process, which induces cross-linking of the material when kept at this temperature for a long time. Indeed, polyamides degradation is quite a complex phenomenon [27,32], which may evolve either towards chain scission or towards branching and cross-linking, depending on various environmental factors [32,33]. In particular, it has been found that at relatively lower temperatures degradation is mostly by cross-linking, while at higher temperatures, chain scission seems to be the main result [34]. Notice anyway that degradation occurs mostly on the R material, rather than V, due to the higher presence of -NH_2_ chain ends in R, consequence of its first degradation occurring during previous recycling. The presence of these chain ends is the key intermediate step that leads to branching and subsequent cross-linking of the polymer chains according to [35,36].

### 3.3. Porosity and Stereomicroscope Analysis

The void percentage was calculated through Equation (1), and the values are shown in Table 4. The results are presented as a function of the raster angle of the specimens, since a certain dependence on this parameter is often observed [8]. On the other hand, concerning this research, the values are around 5% for all samples, with the sole exception of V at 0°. All void percentage results have a low standard deviation, indicating good printing quality and process repeatability. The slight decrease in void percentage for the R material is most probably related to the decrease in the relaxation time and viscosity of the R material, which helps in filling the voids during printing.

**Figure 8:**
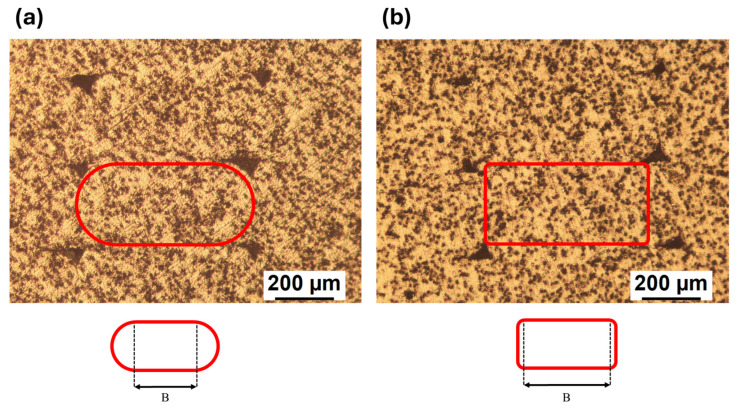
Representative transversal cross sections for (**a**) V and (**b**) R, where B is the base of the deposited line.

The polished transversal cross sections observed at the stereomicroscope are shown in Figure 8 for the V (Figure 8a) and R (Figure 8b) materials. The presence and spatial distribution of porosities are visualized clearly. In both cases, a homogeneous distribution of roughly triangle shaped voids is observed in the contact areas between subsequent deposited lines, which is typical for material extrusion-based 3D printing. The voids of V (Figure 8a) are larger than those seen for R (Figure 8b). In addition, the typical elliptical shape can be observed for V (Figure 8a), while R (Figure 8b) shows a more compact and squared shape. As a consequence, R seems to better fill the available room, spreading more evenly.

Interestingly, each deposited line of both materials is characterized by about the same height, which is reasonable as the layer height is one of the superimposed processing parameters. The main differences between V and R are related to the general shape and the size of the line base (B), which can be inferred from the straight line connecting two adjacent voids (Figure 8). At least 20 line bases were observed at the microscope, and a base length equal to 0.47 ± 0.05 mm was measured for V while 0.59 ± 0.03 mm was obtained for R.

**Figure 9:**
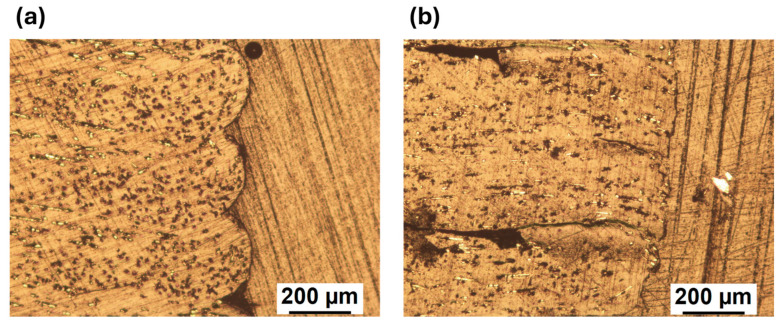
Representative cross section of 90° samples for (**a**) V and (**b**) R.

Additional information can be obtained from the polished cross sections of 90° samples of both materials (Figure 9), examined at the side edge. In the case of V (Figure 9a), the adjacent layers are characterized by a rounded end, while the R material (Figure 9b) has a squared shape. Furthermore, it can be seen that the layers of V are self-supporting (Figure 9a), while those of R tend to lean downward (Figure 9b). This behavior is in line with the lower viscosity of R found in the rheological analyses (Figure 4).

### 3.4. Mechanical Characterization

**Figure 10:**
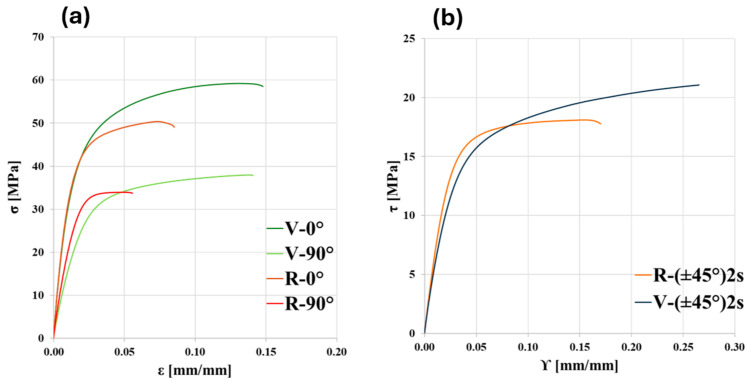
Comparison of representative stress-strain curves of V and R for (**a**) longitudinal and transverse directions and (**b**) shear.

Representative stress vs. strain curves of V and R are compared in Figure 10a for longitudinal and transverse directions and in Figure 10b for shear. All materials are anisotropic, as there are clear differences among the longitudinal, transverse, and shear behavior of both materials, although in all cases, an elasto-plastic behavior is observed. As expected, longitudinal properties are higher than transverse ones for all materials, and this is related to the alignment of reinforcing fibers in the deposition direction [8,15,18,19]. The longitudinal curves of V and R overlap in the initial linear elastic region (Figure 10a). The 90° curve of R has a higher initial slope than that of V; thus, the transverse stiffness is greater (Figure 10a). There is also a certain increase in the yield strength of R, but not as much as the one of stiffness. A similar trend is also observed in Figure 10b, where R has higher shear properties than V, both in terms of stiffness and strength. The main difference that is noticeable is that the R material is considerably more brittle than V. This embrittlement is clearly due to the molecular weight decrease induced by the recycling process, as discussed in Section 3.1 and Section 3.2 above. It can be especially seen in transverse and shear samples, i.e., in the directions where the matrix contributes more to the mechanical properties of the composite.

The significant decrease in strain at break is confirmed quantitatively from the values listed in Table 5, where numerical results obtained from longitudinal, transverse, and shear characterization are reported. In terms of stiffness, $E_{1}$ does not change between V and R because the reinforcement fibers are mostly responsible for the mechanical properties of the composite in the longitudinal direction. Since at 90° and ±45° orientations the behavior depends by a greater amount on the matrix, $E_{2}$ and $G_{12}$ increase by 40% and 25%, respectively, in going from to V to R. The same behavior is observed for yield strength. These results show that in general, R has mechanical properties that are higher than V.

This behavior is well known in the scientific literature, as it has been reported in many papers [10,11,12,14]. Actually, the most striking feature of it is that the recycled material in the form of filament is often weaker than the virgin one [9,11] as a consequence of the partial degradation of the recycled material. Nevertheless, when the recycled material is printed, its mechanical properties exceed those of the virgin one. This phenomenon was first observed by Ding et al. [10], who motivated it through a better impregnation of the carbon fibers by the PA matrix. Lohr et al. [11] and Vidakis et al. [12] noticed the same behavior and justified it based on a plasticization-like effect, while Bandinelli et al. [14] ascribed it to a better wettability and voids content reduction. Interestingly, it is evident in this last paper that such an effect is more pronounced in the tubular specimens (i.e., in the transverse direction) than in the longitudinal direction. This is exactly what was found in the case of the present paper. This makes it clear that this effect must be explained based on the matrix mechanical behavior.

In fact, void content cannot explain the observed phenomenon. The main void percentage reduction in going from V to R (about 35%) is in the longitudinal direction, where fiber mechanical behavior is dominant; therefore, the reason for the transverse and shear properties increase from V to R must be looked for elsewhere. Matrix crystallinity may be a suitable explanation, because it is common that a lower molecular weight induces a higher crystallinity, because of kinetic crystallization reasons. On the other hand, also this hypothesis is not convincing, since in the case of the present paper, crystallinity percentage is low and about equal for V and R (Table 3). Indeed, despite more rapid crystallization kinetics observed in the case of R, both materials can be regarded as essentially amorphous.

**Figure 11:**
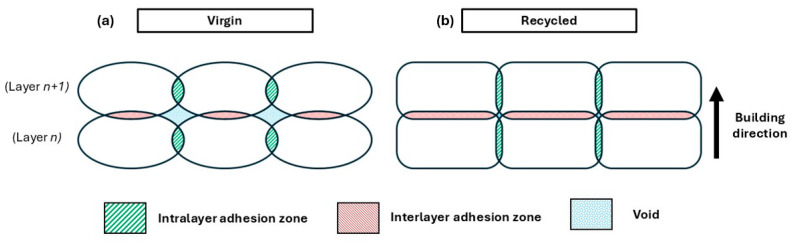
Adhesion zones and voids distribution in (**a**) V and (**b**) R.

The most likely explanation is sketched in Figure 11, where schemes of the transverse cross section of V (Figure 11a) and R (Figure 11b) are shown. Low molecular weight determines a lower viscosity (Figure 4a,b), and for the same printing parameters, the R material is more fluid. Thus, the deposed 3D printed lines have a better spreadability onto the previously deposed material. This is in agreement with the observations made from the stereomicroscope surface analysis (Figure 8 and Figure 9), where R appears to be more compact and the contact surfaces are larger. This can be seen both in the interlayer areas (i.e., between one layer and the next one, red areas in Figure 11), and intralayer areas (i.e., between one deposited line and the next one, green areas in Figure 11).

Moreover, when new molten material is extruded, it encounters the already deposited one, both in the adjacent lines and on the previously deposed layer. This material, then, undergoes a temperature increase, which leads to a partial remelting [37]. In this state, molecular diffusion occurs, and once cooled, welding is achieved. Both interlayer and intralayer adhesion strength are closely related to the weld strength [38,39], which in turn is closely related to molecular diffusion at the interface [40,41,42]. This is enhanced by the lower molecular weight of R that favors self-diffusivity. In fact, it is well known from the literature that the chain self-diffusion coefficient in an entangled polymer melt generally increases with the decrease in molecular weight. This is a fundamental result of reptation theory, which predicts that self-diffusion is inversely proportional to the square of the average molecular weight [43,44]. Recent studies tackled this issue from an experimental [45,46] and numerical point of view [47,48,49], and a dependence with slightly different exponent is normally found, yet it is well understood that polymer chains of low molecular weight self-diffuse faster. Moreover, self-diffusivity is also enhanced by the mainly amorphous nature of the matrix material, as crystallinity does not exceed 20%, thus making it more prone to the diffusion of smaller molecules.

**Figure 12:**
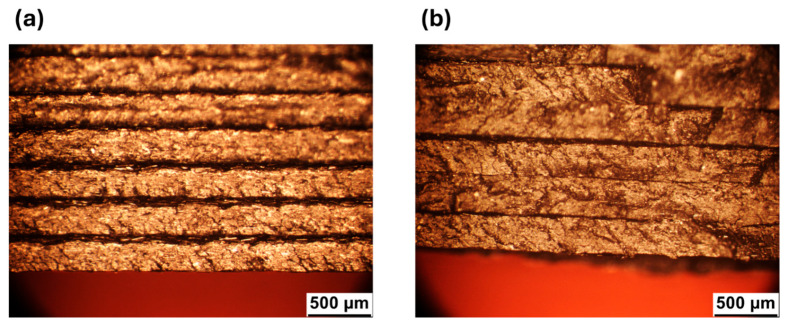
Fracture surface from 90° sample for (**a**) V and (**b**) R.

The behavior described so far is confirmed by the fracture surface obtained from the samples at 90° (Figure 12). In the case of V (Figure 12a), a ductile fracture can be observed, and the successive layers are clearly visible. In contrast, R (Figure 12b) exhibits a brittle fracture, with a more compact section and layering that is less distinct than in V.

## 4. Conclusions and Future Developments

In this paper, the mechanical recycling of FDM 3D printing short carbon fiber reinforced polyamide was investigated concerning the possibility of grinding it and reobtaining a filament that can be used for further 3D printing. Such a topic is of relevant and actual importance, since it enhances its eco-sustainability and circular economy. V and R materials were compared to each other concerning their thermal, rheological, and tensile mechanical properties. The rheological testing indicated quite clearly that the process of grinding and remelting the material induced a decrease in the molecular weight of the material, as shown by a sensible reduction in the complex viscosity.

Unexpectedly, transversal strength and stiffness of the R printed parts were not in line with such a molecular weight reduction. The explanation for such behavior could not be ascribed to an increase in material crystallinity nor a void content reduction. Moreover, fiber orientation should not play a significant role, since the longitudinal properties are almost identical between V and R.

The most likely explanation, thus, was proposed to be linked to an increase in the interlayer and intralayer contact area and intralayer and interlayer adhesion during printing. Indeed, the lower molecular weight of R does induce an increase in self-diffusivity, which in turn leads to better interlayer and intralayer welding, and this has a clear effect on the mechanical properties. Notice that the effect is indeed more pronounced on the transverse and shear properties, rather than the longitudinal ones, because welds are mostly loaded only when the material is stressed in these directions. As a result, one can conclude that an ideal material for 3D printing based on material extrusion should have a molecular weight distribution that comprises both a high molecular weight fraction for allowing extrusion, and a low molecular weight fraction for ensuring good welding quality. These two characteristics taken together allow good mechanical properties of the printed part.

The results that are shown in this paper are concerned with a single reprocessing stage. Increasing the number of reprocessing stages, molecular weight decreases more, thus making the filament extrusion more difficult, and even in the case that 3D printing was successful, brittleness would increase, leading to lower mechanical properties unless suitable countermeasures are taken, such as mixing with a certain amount of virgin material or adding toughening agents.

## Figures and Tables

**Table 1:** Material properties from technical datasheet.

Density [g/cm^3^]	Young’s Modulus [GPa]	Yield Stress [MPa]	Strain at Break [mm/mm]	HDT [°C]	Impact Strength [J/m]
1.2	2.4	40	0.25	145	330

**Table 2:** Printing parameters for V and R materials.

Parameter [Unit]	Value
First layer height [mm]	0.2
Layer height [mm]	0.3
Line width [mm]	0.4
External shells	0
Top and bottom layers	0
Infill [%]	100
Infill pattern—Rheology	Concentric
Infill pattern—Tensile	Unidirectional
Infill raster angle—Tensile	0°, 90°, [±45°]_2s_
Printing speed [mm/s]	30
Extrusion temperature [°C]	250
Plate temperature [°C]	70

**Table 3:** Results of DSC thermal characterization.

	V			R	
DSC Phases	Peak Temp [°C]		$\chi_{c}$ [%]	Peak Temp. [°C]	$\chi_{c}$ [%]
1° Heating	199.9 (1.5)		22.4 (1.8)	201.0 (1.0)	16.3 (0.3)
Cooling	162.6 (0.4)		21.2 (1.8)	169.5 (0.3)	15.5 (2.2)
2° Heating	194.8 (0.2)	201.0 (0.6)	19.0 (0.2)	199.6 (0.8)	16.4 (0.8)

**Table 4:** Void percentage as a function of raster angle; values in round brackets represent standard deviation.

Material	V			R		
Raster angle	0°	90°	±45°	0°	90°	±45°
v [%]	6.7 (0.9)	5.0 (0.9)	5.8 (0.8)	4.6 (0.8)	4.3 (0.4)	4.7 (0.2)

**Table 5:** Results of mechanical characterization from tensile tests. Values in round brackets represent standard deviation.

Mechanical Property	V	R
$E_{1}[GPa]$	3.68 (0.06)	3.83 (0.06)
$E_{2\;}[GPa]$	1.64 (0.15)	2.27 (0.03)
$\nu_{12}$	0.40 (0.02)	0.33 (0.03)
$G_{12\;}[GPa]$	0.60 (0.04)	0.74 (0.04)
$\sigma_{Y}^{0{}^{\circ}\;}[MPa]$	43.70 (0.45)	42.72 (1.12)
$\sigma_{Y}^{90{}^{\circ}\;}[MPa]$	27.56 (1.79)	32.21 (0.94)
$\tau_{Y}\;[MPa]$	12.62 (0.48)	15.19 (0.76)
$\varepsilon_{ul}^{0{}^{\circ}}\;[mm/mm]$	0.14 (0.02)	0.09 (0.02)
$\varepsilon_{ul}^{90{}^{\circ}}\;[mm/mm]$	0.15 (0.02)	0.05 (0.01)
$\gamma_{ul}\;[mm/mm]$	0.29 (0.05)	0.16 (0.02)

## Data Availability

The original contributions presented in this study are included in the article. Further inquiries can be directed to the corresponding author.
